# Supplementary material for: Content-rich biological network constructed by mining PubMed abstracts
Source: BMC Bioinformatics. 2004 Oct 8;5:147. doi: 10.1186/1471-2105-5-147 (PMC528731; doi:10.1186/1471-2105-5-147)
Supplement: Additional File 5 — The original Chilibot query results of the term "long-term potentiation (LTP)" and 22 other terms, limiting the latest references analyzed to the years 1990, 1995, 2000, and 2004. [file 1471-2105-5-147-S5.bz2 › chilibotAdditionalFile5/ltp2004/html/LTP_CREB.html]

 


 **LTP** and **CREB** 
  
Found 64 abstracts in PubMed,  **30 abstracts were retrieved and analyzed**.  


---

 Search Google  |
 PDF files only 
|  EDU domain only 

---

**Interactive relationship** (e.g. stimulation, inhibition, etc)

- Expression of constitutively active  **CREB**  protein facilitates the late phase of long term potentiation  [ **LTP** ]  by enhancing synaptic capture.  Ref: 11893339 Cell, 2002
- **LTP**  was accompanied by increased protein synthesis and increased phosphorylation of  **CREB**  in entorhinal cortex, effects that were also blocked by genistein.  Ref: 12099488 Hippocampus, 2002
- Acute lithium treatment for 2 days also enhanced  **LTP** , which was accompanied by the elevated expression of p  **CREB** , but not by neurogenesis.  Ref: 12716419 J Neurochem< MedlineTA>J Neurochem, 2002
- However, a brief treatment with forskolin, which is effective for short term potentiation and which could also activate  **CREB** , was not sufficient to induce long term potentiation  [ **LTP** ]  of resealing.  Ref: 12529429 Mol Biol Cell, 2003
- Pharmacological and two pathway experiments suggest a model in which VP16  **CREB**  activates the transcription of CRE driven genes and leads to a cell wide distribution of proteins that prime the synapses for subsequent synapse specific capture of L  **LTP**  by a weak stimulus.  Ref: 11893339 Cell, 2002
- L  **LTP**  can be induced by either multiple train tetanization, NO or 8 Br cGMP paired with one train tetanization, or the cAMP activator forskolin, and all three types of potentiation are accompanied by an increase in phospho  **CREB**  immunofluorescence in the CA1 cell body area.  Ref: 12205148 J Neurophysiol, 2002
- Restricted and regulated expression in mice of VP16  **CREB** , a constitutively active form of  **CREB** , in hippocampal CA1 neurons lowers the threshold for eliciting a persistent late phase of long term potentiation  [ **LTP** ]  L  **LTP**  in the Schaffer collateral pathway.  Ref: 11893339 Cell, 2002
- Here, we briefly review experiments that have shown MAPK ERK, cAMP response element binding protein  **CREB**  and the immediate early gene IEG zif268 are essential components of a signalling cascade required for the expression of late phase  **LTP**  and of certain forms of long term memory.  Ref: 12740127 Philos Trans R Soc Lond B Biol Sci, 2003

**Parallel relationship** (e.g. studied together, co-existance, homology, etc.)

- ... an injection of oxytocin antagonist in multiparous mice significantly inhibited the improved spatial memory, L  **LTP**  and  **CREB**  phosphorylation.  Ref: 12598900 Nat Neurosci, 2003
- These observations suggest that Alphabeta acts directly on the pathways involved in the formation of late  **LTP**  and agents that enhance the cAMP PKA  **CREB**  signaling pathway have potential for the treatment of AD.  Ref: 12244210 Proc Natl Acad Sci U S A, 2002
- Amyloid beta peptide inhibition of the PKA  **CREB**  pathway and long term potentiation  [ **LTP** ]  reversibility by drugs that enhance cAMP signaling.  Ref: 12244210 Proc Natl Acad Sci U S A, 2002
- An increase in  **CREB**  phosphorylation and L  **LTP**  induced by one train tetanus were observed in the multiparous mouse hippocampus without oxytocin application.  Ref: 12598900 Nat Neurosci, 2003
- In oxytocin perfused hippocampal slices, one train tetanus stimulation induced long lasting, long term potentiation  [ **LTP** ]  L  **LTP**  and phosphorylation of cyclic AMP responsive element binding protein  **CREB** , and MAP kinase inhibitors blocked these inductions.  Ref: 12598900 Nat Neurosci, 2003
- The facilitation of hippocampus based, long lasting synaptic plasticity, which is frequently investigated in model systems such as long term potentiation  [ **LTP** ]   **LTP**  and in learning paradigms such as the Morris water maze, is associated with several cellular key events calcium influx through the N methyl D aspartate NMDA receptor, generation of cyclic AMP cAMP and activation of protein kinase A PKA, phosphorylation of mitogen associated protein kinase MAPK and cAMP response element binding protein  **CREB** , and subsequent transcription of plasticity associated genes.  Ref: 12668903 Mol Neurobiol, 2003
- A recent study using mice carrying a mutation in a specific tyrosine residue of trkB pinpoints a necessary role for the phospholipase Cgamma pathway in  **CREB**  activation and  **LTP**  maintenance.  Ref: 12689763 Trends Neurosci, 2003
- These results suggest that NO, cGMP, and PKG cause release of calcium from ryanodine sensitive stores, which in turn causes phosphorylation of  **CREB**  in parallel with PKA during the induction of L  **LTP** .  Ref: 12205148 J Neurophysiol, 2002
- Ryanodine receptors contribute to cGMP induced late phase  **LTP**  and  **CREB**  phosphorylation in the hippocampus.  Ref: 12205148 J Neurophysiol, 2002
- Moreover, blockade of NMDA receptor NMDAR did not decrease FAK, MAPK ERK, and  **CREB**  phosphorylation although  **LTP**  induction was blunted by NMDAR antagonist.  Ref: 12764094 J Neurosci, 2003
- We previously found that the nitric oxide NO cGMP cGMP dependent protein kinase PKG signaling pathway acts in parallel with the cAMP cAMP dependent protein kinase PKA pathway to produce protein and RNA synthesis dependent late phase long term potentiation  [ **LTP** ]  L  **LTP**  and cAMP response element binding protein  **CREB**  phosphorylation in the CA1 region of mouse hippocampus.  Ref: 12205148 J Neurophysiol, 2002
- These results imply that long term potentiation  [ **LTP** ]  by  **CREB**  required activation by another, cAMP independent pathway.  Ref: 12529429 Mol Biol Cell, 2003
- As both  **CREB**  phosphorylation and EGR1 induction have been linked to long term potentiation  [ **LTP** ]  and some forms of memory consolidation, these results may implicate  **CREB**  and EGR1 in independent or partially independent cholinergic signalling pathways involved in memory processes.  Ref: 12124440 J Neurochem, 2002
